# Incidental Discovery of a Chronically Dissected Pulmonary Artery

**DOI:** 10.7759/cureus.92095

**Published:** 2025-09-11

**Authors:** Justin Lee, Olivia J He, Aashi Saraf, Karthik Kanamalla

**Affiliations:** 1 Radiology, SUNY Downstate College of Medicine, Brooklyn, USA; 2 Pulmonary and Critical Care, NewYork-Presbyterian (NYP) Brooklyn Methodist Hospital, Brooklyn, USA; 3 Radiology, SUNY Downstate Health Sciences Center, Brooklyn, USA

**Keywords:** chronic dissection, chronic pulmonary hypertension, internal med, pulmonary artery dissection, pulmonary critical care, pulmonary trunk

## Abstract

Pulmonary artery dissection is a rare and potentially fatal vascular emergency most often associated with pulmonary hypertension. In this report, we describe the incidental discovery of a chronically dissected pulmonary artery in a 92-year-old woman who presented to the emergency department with abdominal discomfort, constipation, and poor appetite. Imaging to evaluate for occult malignancy revealed a dissection flap within the main pulmonary artery and findings suggestive of chronicity. These radiographic findings included parenchymal scarring of the left lung, calcification, and collateralization of pulmonary vessels. Given the patient’s age and lack of symptoms, no invasive treatment approach was pursued after multidisciplinary discussion. Classically considered a deadly disease, pulmonary artery dissection may not be as universally fatal as once thought and may present subclinically, remaining undetected for extended periods of time. Early recognition of atypical and indolent presentations is essential, especially in an era of widespread cross-sectional imaging and in patients for whom invasive intervention may not be appropriate.

## Introduction

Pulmonary artery dissection is an emergent and life-threatening condition that is exceptionally rare, albeit increasingly reported in the literature with growing use of cross-sectional imaging [1]. Dissection occurs when there is propagation of a longitudinal tear through the arterial intima into the mid or deep media, resulting in a perfused true and nonperfused false lumen. Adobo et al. have organized the causes of pulmonary artery dissection into four major groups: (1) congenital malformation, (2) infection or inflammation, (3) acquired cardiac disease, and (4) iatrogenic causes [2]. Of these, pulmonary hypertension is most often cited as an underlying risk factor [3]. A challenge complicating the diagnosis of this rare entity is that it presents with non-specific symptoms. Most commonly, dyspnea, retrosternal chest pain, and cyanosis are present [4]. As with dissections of the aorta, pulmonary artery dissection can often be fatal if left untreated, resulting in obstructive and cardiogenic shock. Natural progression of a dissection tends to cause arterial rupture and results in hemopericardium, hemomediastinum, hemothorax, pulmonary hemorrhage, and eventual cardiac tamponade [5].

In this paper, we describe the incidental discovery of a dissected pulmonary artery in an asymptomatic elderly woman. While there are four other documented cases of asymptomatic dissection in the literature, this report is unique in that radiographic findings support a diagnosis of long-standing, chronic pulmonary artery dissection - again challenging the notion that this disease is invariably and rapidly fatal [6].

## Case presentation

A 92-year-old female with a past medical history of hypertension, hyperlipidemia, and stenting for coronary artery disease presented to our institution’s emergency department for the evaluation of two weeks of intermittent abdominal pain, nausea, and bloating. History was notable for poor appetite and constipation. She denied having shortness of breath, chest pain, diarrhea, bloody or black stools, hematuria, dysuria, or fever. The patient was a non-smoker and had no pertinent family history. A focused physical examination was unremarkable - no abdominal distention, no tenderness to palpation, normoactive bowel sounds, and no cyanosis. Vital signs were within normal limits. She appeared to be in no acute distress. There was no family or social history pertinent to the presenting complaint. Her prescribed home medications included aspirin, metoprolol succinate, and rosuvastatin.

Initial evaluation with basic metabolic profile and complete blood count was significant for hyponatremia, hypochloremia, and low serum bicarbonate (Table 1). Venous blood gas, hepatic function panel, and urinalysis were within normal limits. Computed tomography (CT) of the abdomen and pelvis was then performed after the administration of intravenous contrast. No acute intra-abdominal process was identified, but an incidental note was made of partially thrombosed 3 cm aneurysms of the right common and internal iliac arteries, and left internal iliac artery. The left common iliac artery was ectatic.

**Table 1:** Serum and Urine Chemistry on Admission Note that there are no established reference ranges for urine electrolytes.

Serum	Value (mmol/L)	Reference range (mmol/L)
Na^+^	123	136 – 146
K^+^	4.3	3.5 – 5.0
Cl^–^	89	98 – 106
HCO_3_^–^	20	24 – 31
Urine		
Na^+^	143	–
K^+^	29.8	–
Cl^–^	138	–
Osmolality	Value (mOsm/kg)	Reference range (mOsm/kg)
Serum	260	275 – 295
Urine	468	300 – 1,000

The patient was admitted to the hospital for workup and treatment of hyponatremia and constipation. Serum osmolality, urine sodium, and urine osmolality suggested syndrome of inappropriate antidiuretic hormone secretion (SIADH), hypothyroidism, or adrenal insufficiency as potential underlying causes. The latter two were ruled out after obtaining thyroid function tests and serum cortisol levels. As SIADH is often associated with lung disease, most commonly malignancy, a contrast-enhanced CT of the chest was performed.

**Figure 1:**
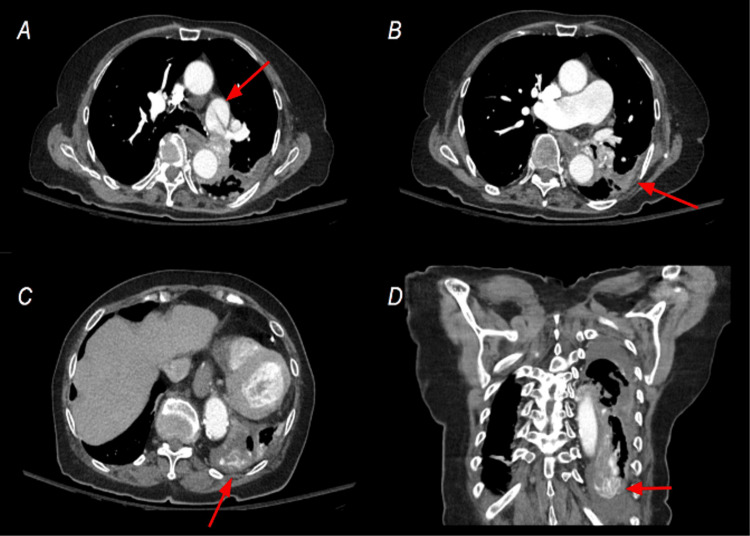
Pulmonary Artery Dissection on Contrast-Enhanced Chest CT A dissection flap within the main pulmonary artery (A) appears to extend into the lobar artery of the left lower lobe, resulting in thrombus and occlusion. Parenchymal disease in the left lower lobe (B) and a relatively dilated right main pulmonary artery can suggest either increased compensatory flow to a non-diseased right lung or underlying pulmonary artery hypertension. Chronicity of this finding was further supported by contrast-filled collateral vessels supplying the inferior portion of the left lower lobe (C,D).

**Figure 2:**
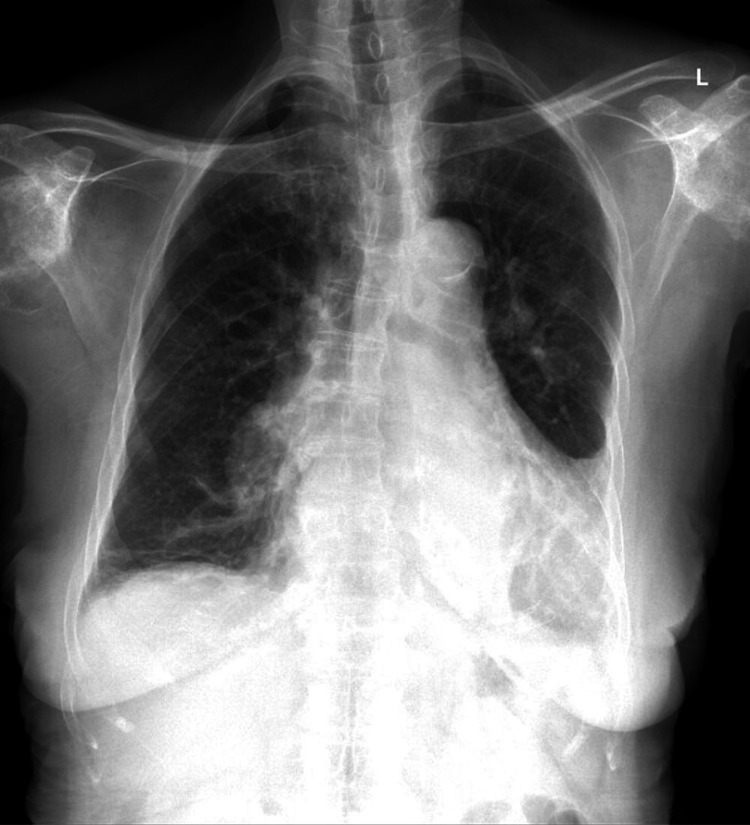
Pre-admission Chest Radiograph Pre-admission chest radiograph notable for nonspecific airspace disease within the left lower lung, retrocardiac area. Although more evident in retrospect, the right pulmonary, interlobar, and right lower lobar arteries appear dilated. This was concordant with findings on CT.

CT chest demonstrated a dissection flap within the main pulmonary trunk which was suspected to involve an occluded left lower lobar artery (Figure 1). Atelectasis, dystrophic calcification and parenchymal scarring, and volume loss present in the left lower lobe suggested chronicity. The presence of numerous contrast-filled collateral vessels further supported this hypothesis. The main pulmonary trunk and right pulmonary artery and distal branches were dilated - a finding associated with pulmonary arterial hypertension, or a sequela of increased compensatory flow to the right lung. Suggestion of a dilated interlobar artery was also present on pre-admission plain radiography (Figure 2).

After discussions with vascular and cardiothoracic surgeons, and based on the patient’s age, lack of symptoms, and perceived chronicity of radiographic findings, it was decided that no invasive management would be pursued for either her iliac artery aneurysms or pulmonary artery dissection. Her hyponatremia resolved after treatment with fluid restriction, salt tablets, low-dose furosemide, and optimization of nutritional status. An echocardiogram was performed one day prior to discharge for the evaluation of pulmonary hypertension which yielded a pulmonary artery systolic pressure of approximately 39 mmHg. Additional investigation with right heart catheterization was not pursued. She was discharged uneventfully with appropriate outpatient follow-up.

## Discussion

Pulmonary artery dissection is a rare complication typically associated with chronic pulmonary hypertension. An apparent high mortality is based on limited data. Its lethality was felt to undermine its true prevalence as most cases were historically identified at autopsy [2]. However, this has changed - since its initial description by Helmbrecht et al. in 1842, approximately 90 cases have been reported in patients who were alive at the time of diagnosis [6]. Increased ante-mortem diagnosis is the direct result of widespread use of cross-sectional diagnostic imaging, particularly in the emergency setting. Other modalities, such as echocardiography and catheter-directed angiography, have also been used to evaluate for dissection of the pulmonary artery [1,7].

Unlike dissection of large systemic arteries, which tend to result in re-entry tears and equalization of false and true lumen pressures, pulmonary artery dissection tends to rupture, resulting in cardiogenic shock and sudden death [2]. Rupture can occur into the pericardium, mediastinum, lungs, or pleural cavity [8]. This pathophysiological difference is thought to be secondary to the inherent low resistance of the pulmonary circulation and a thinner, relatively more fragile arterial media [1].

As far as the underlying etiology of pulmonary artery dissection, four major categories have been described - congenital malformation, infection or inflammation, acquired cardiac disease, and iatrogenic causes [2]. Pulmonary hypertension is reported as the most common underlying condition, predisposing to cystic medial necrosis [9]. Other causes described in the literature that fall into these broad categories include patent ductus arteriosus, infective endocarditis, Eisenmenger syndrome, chronic obstructive pulmonary disease, and catheter-induced vessel wall injury [6,9]. In some patients, the cause is unknown [10]. In this 92-year-old woman, an echocardiogram was performed prior to discharge to evaluate for pulmonary hypertension as a potential underlying cause. Pulmonary artery systolic pressure was, in fact, elevated at 39 mmHg. However, it is unclear whether her pulmonary hypertension preceded the dissection or developed as a result of chronic dissection and parenchymal disease.

No general consensus has been reached regarding optimal management of pulmonary artery dissection, likely a byproduct of its rarity [6]. Medical management commonly includes a combination of vasodilator agents, diuretics, and beta-adrenergic blockers [6]. These parallel the treatment protocol used in dissections of the aorta. Others suggest surgery as the most appropriate treatment, especially in symptomatic and critical patients [11,12]. In one study, concomitant aortic and pulmonary artery dissections occurring in the setting of a patent ductus arteriosus were treated with endovascular stent-grafting of the aorta with persistence of a dissected pulmonary artery [13]. Still, some authors suggest a combination of both medical and surgical treatment should be employed [14]. A more recent analysis of 73 cases found that surgical intervention was successful in 90.3% of cases, indicating that invasive treatment may be most optimal [6]. It is equally important to consider early detection and appropriate treatment of heart and lung disease as a form of primary prevention.

In our literature review, we identified four reported cases of pulmonary artery dissection that were either asymptomatic or incidentally discovered, and therefore hypothesized to be chronic. These included 17- and 23-year-old males who had undergone prior pulmonary balloon valvuloplasty, a 62-year-old woman with ventricular septal defect and Eisenmenger’s syndrome, and a 97-year-old female with chronic heart failure [6,7,15-17]. A unique aspect of our patient’s case - and not present in prior reports - is with regard to CT imaging findings, which provide specific, concrete evidence of chronic disease. Since the patient had not reported any recent or remote history of characteristic symptoms, it is suggested that the dissection had occurred silently. This again supports the growing body of evidence that this condition can be survivable.

## Conclusions

Pulmonary artery dissection is an exceptionally rare disease with often poor prognosis. However, more recent evidence suggests that this condition is treatable and survivable. This is especially the case given the increased utilization of diagnostic imaging in acute care settings. Yet it is still important to recognize the potential for rapid decompensation, cardiogenic shock, and sudden death in patients with pulmonary artery dissection. Detection and treatment of risk factors, especially pulmonary hypertension, can help to reduce the risk of its development. 
